# Treatment seeking delay and associated factors among tuberculosis patients attending health facility in Ethiopia from 2000 to 2020: A systematic review and meta analysis

**DOI:** 10.1371/journal.pone.0253746

**Published:** 2021-07-01

**Authors:** Mohammed Suleiman Obsa, Wakgari Binu Daga, Naol Gorde Wosene, Tsegaye Demeke Gebremedhin, Dinkisisa Chemeda Edosa, Abdurahman Tune Dedecho, Nefsu Awoke, Bedilu Girma Weji, Eyob Eshetu Bekele

**Affiliations:** 1 School of Anesthesia, Wolaita Soddo University, Wolaita Soddo, Ethiopia; 2 School of Public Health, Wolaita Soddo University, Wolaita Soddo, Ethiopia; 3 Department of Anesthesia, Arsi University, Wolaita Soddo, Ethiopia; 4 School of Nursing, Wolaita Soddo University, Wolaita Soddo, Ethiopia; 5 Department of Anesthesia, Saint Paul’s Hospital Mellinium Medical College, Addis Ababa, Ethiopia; 6 School of Vetirnary Medicine, Wolaita Soddo University, Wolaita Soddo, Ethiopia; The University of Georgia, UNITED STATES

## Abstract

**Background:**

Treatment seeking delay is defined as the time interval between the onset of the major symptoms of tuberculosis (TB) and the first visit to the formal health care facility. The patient was said to be delayed if the patient visited the health-facility after 3 weeks onset of major symptoms. However, in low-income countries like Ethiopia, the delay in treatment-seeking among tuberculosis patients contributes to a widespread transmission and high prevalence of tuberculosis.

**Methods:**

Studies were retrieved from PubMed, Cochrane Database, Cinahl, Scopus, Mednar, and Google Scholar by employing a combination of search terms with Boolean operators. Heterogeneity across studies was assessed using the Cochrane Q test. A funnel plot was used for visual assessment of publication bias. Subgroup analyses were performed to explore the possible causes of heterogeneity. Egger’s weighted regression test at a p-value < 0.05 was used to assess the presence of publication bias. Sensitivity analysis was performed to judge whether the pooled effect size was influenced by individual study. STATA software version 14 was used for all statistical analyses.

**Result:**

A total of 12 studies with 5122 total sample size were included. The national pooled prevalence of treatment seeking delay was 44.29% (95% CI: 39.805, 48.771). The visual inspection of the funnel plot showed the asymmetrical distribution, and the Egger test showed insignificant (P = 0.348). Patients who did not seek formal health care providers on a first contact had about 7 times more likely to delay than patients who sought formal health care provider on a first contact (OR: 7.192 ((95% CI 5.587–9.257), P = 0.001, I^2^: 85%). The others independent predictors of delay were rural residence (OR: 3.459 ((95% CI 1.469–8.148), P ≤ 0.001), extra pulmonary TB (OR: 2.520 ((95% CI 1.761–3.605), 0.180), lower educational level (OR 11.720 ((95% CI 1.006–2.938), P <0.001), and distance more than 10km from health facility (OR: 1.631 ((95% CI (10.858–3.101), P = 0.001).

**Conclusion:**

In this review, we identified a substantial treatment seeking delay among TB patients in Ethiopia. And, the independent predictors of delay were treatment sought before formal health care provider, residence of the patient, type of TB, educational level, and distance from a health facility. Thus, we recommend health extension workers, health professionals and other stakeholders to focus on patient education, and to continuously mobilize the whole communities on early treatment seeking with a special emphasis given to where treatment sought before formal health care provider, rural resident, extra pulmonary TB, and a patient living farther than 10km distance from health facility.

## Background

Tuberculosis (TB) is a communicable disease that can be mainly caused Mycobacterium tuberculosis. It can also be rarely caused by Mycobacterium bovis and Mycobacterium africanum [1]. TB infected a third of the world’s population each year [2,3]. Globally, 10.4 million incident cases, of whom 6.1million notified and 1.4 million deaths were estimated to occur in 2015 [4]. Thus, the latest strategy, “End TB” has been designed in line with the sustainable development goals (SDG). The strategy aimed at reaching 90% case detection and treatment success by 2025 [5]. According to the 2020 globally milestones of the ‘End TB Strategy’, the annual reduction of TB prevalence should be by a rate of 4–5% [6,7]. However, Ethiopia is still one among the 30 high burdens TB countries and The TB case detection rate is very low compared to the World Health Organization (WHO) target of detecting all infectious TB cases [8]. A recent study conducted in Ethiopia showed that more than half of TB patients reported to have a treatment seeking delay [9].

There is no standard definition of treatment seeking delay. Different studies used different definitions of delay. Almost all studies defined “treatment seeking delay” contextually as the time interval between the onset of the major symptoms of TB to the first visit to formal health care facility [10–12]. TB transmissions occur between onset of symptoms and initiation of treatment. Hence, early case detection and timely initiation of the treatment are the basis to reverse the incidence of the disease [13,14].

Although most patients can be cured with a timely diagnosis and early treatment initiation, the delay in treatment seeking is a major concern in most low-income countries [15,16]. The main barrier is the detection of sputum smear-positive TB cases which depends on passive case findings and self-reports to the health facilities. This is highly dependent on patient inspiration, financial capability, degree of suspiciousness of health workers, and the accuracy and effectiveness of diagnostic services [15]. Similarly, a study found that more than half of the patients perceived as symptoms would disappear without treatment while more than quarter of them seek traditional treatment [15,17].

In Ethiopia, almost 50% of all suspected pulmonary tuberculosis patients had a history of treatment seeking delay [18]. Hence, the remaining half will continue to transmit TB infection in the community until detected and treated by another health care provider [19,20]. Furthermore, a single untreated case of smear positive tuberculosis can infect up to 15 individuals annually and over 20 during the natural course of disease [21].

The goal of TB control programs is to reduce transmission within the community. Achieving this goal requires a considerable time because most persons in endemic areas are already infected, constituting a pool that continuously contributes to the source of infectious cases. Delay in treatment seeking is significant not only due to disease prognosis at the individual level, but also due to transmission within the community and the reproductive rate of the TB epidemic [22].

Although Ethiopia adopted the Direct Observed Therapy (DOT) program, the disease has still become a major public health problem. On the other hand, the TB case detection rate in Ethiopia was 67.3%, which was lower than the target set by the ministry of health and WHO. Furthermore, this detection rate also highly variable across the region and the district [20].

At the beginning of Millennium Development Goal, our country aimed to reduce TB related mortality by more than 50%. In addition, in 2004, Ethiopia launched Health Extension Workers (HEWs) program with the aim of delivering health information to the community. But, the burden of the disease and poor treatment seeking behavior among TB patient is still now high. This directly affects effective TB control program in the country. There have been also conflicting findings on the prevalence and risk factors of treatment seeking delay. Hence, this review was aimed to solve these conflicting issues by generating the national pooled prevalence of treatment seeking delay and factors affecting delay among tuberculosis patients in Ethiopia.

## Method

The Eligibility criteria based on the Condition, Context and Population (**COCOPO**) principle adapted from JBI 2017 guideline of prevalence review.

**Types of population:** Tuberculosis patients who reported to have a treatment seeking delay, and/or patient delay.

**Context:** This review considered only studies conducted in Ethiopia.

**Condition:** This review considered studies that measured the outcome of interest based prevalence and risk factors of treatment delay.

### Inclusion criteria

All observational studies conducted on patient delay, and/or treatment seeking delays at health facilities in Ethiopia were considered. This systematic review and meta analysis included studies which measured the outcome of interest on prevalence and risk factors of patient delay, and/or treatment seeking delay. Furthermore, studies that explained a two by two table of frequency of independent variable with a dependent variable to determine factors affecting treatment seeking delay were included. In addition, the studies that used 3 weeks or 21days as a cutoff point to determine presence or absence of treatment seeking delay were included. Published and unpublished studies written in English language from January 01, 2000 to October 24, 2020 were also considered.

### Exclusion criteria

Studies that used median to determine treatment seeking delay, and associated factors were excluded. On the other hand, studies that used 15 days and 30 days as a cutoff point to determine treatment seeking delay were excluded. Studies that did not explain a two by two table of frequency of independent variable with a dependent variable to determine factors affecting treatment seeking delay, studies that were not fully accessed, studies that were not conducted on human, studies that did not conduct in English language, studies that conducted before January 01, 2000 and after October 24, 2020, studies that conducted only on health systems delay or studies that did not report patient delay, and/or treatment seeking delay, study with data inconsistence whose author failed to respond after multiple request were excluded. Final exclusion was done after careful critical appraisals. The 2017 Joanna Briggs Institute (JBI) Critical Appraisal instruments for prevalence studies were used to evaluate the methodological quality of each article [23]. The used criteria include: appropriateness of sampling frame to address the target population, adequacy of sample size, detail description of the study subjects and the setting, conduction of data analysis with sufficient coverage of the identified sample, selection of participants in appropriate ways, methods used for the identification of the condition, measurement of the condition in a standard, reliable way for all participants, appropriateness of statistical analysis used, and adequacy of the response rate, and if not, how appropriately the low response rate managed. After evaluation of each study against these criteria, studies with a score of below 7 out of 9 criteria of Critical Appraisal instruments for prevalence studies were excluded. On the other hand, studies with a minimum score of 7 or above were included.

### Study design and search strategy

A search beyond published literature was performed to reduce the risk of publication bias. The search was also restricted to studies published in the English language from January 01, 2000 to October 24, 2020. An initial limited search of PubMed, Cochrane Database, Cinahl, Scopus, Mednar and Google scholar were undertaken for published articles. Unpublished studies were searched from the Addis Ababa University electronic thesis and dissertation, and Jimma University open access institutional repository. These followed by analysis of the text words contained in the title and abstract, and of the index terms used to describe article. A second search using all identified keywords and index terms was then undertaken across all included databases. Thirdly, the reference list of all identified reports and articles were searched for additional studies. We performed the literature search using the medical subject headings (MeSH) and text words related. The following search key terms were used to include studies from above mentioned data base: “Tuberculosis”, “patient delay”, “treatment seeking delay”, "Time-to-Treatment", and “Ethiopia”. The Boolean operators (AND and OR) combination were used to search databases. The PubMed search terms with their Boolean operators of this review was attached as an additional file (**S1 File. Search details**).

### Reporting

This systematic review and meta-analysis followed the Preferred Reporting Items for Systematic Reviews and Meta-Analyses (PRISMA) guidelines for literature search strategy, selection of studies, data extraction, and result reporting [23] (**S2 File. Prisma 2017 Checklist**). The protocol was not registered in the Prospero database.

### The outcome of the study

The outcomes of this review were treatment seeking delay and associated factors.

### Operational definition

#### Treatment seeking delay

The time period from onset of TB symptoms till first presentation to a formal health care facility. The patient was said to be delayed if the patient visited a health-facility after 3 weeks or twenty one days of onset of major TB symptoms.

#### Onset of symptom

It is the day when the patient first becomes aware of the symptom or symptoms.

#### Formal-health care facility

Modern health care facility such as health posts, clinics, health centers and hospitals owned by the government or the private sector.

#### Studies with good methodological quality

Studies with a minimum score of 7 or above upon evaluation against Critical Appraisal instruments for prevalence studies.

#### Studies with poor methodological quality

Studies with a score of below 7 out of 9 criteria upon evaluation against Critical Appraisal instruments for prevalence studies.

#### Non-formal health care facility

It included traditional health care providers and drug retail outlets.

#### Pulmonary tuberculosis

It is a type of tuberculosis that exclusively affects the lung parenchyma.

#### Extra pulmonary tuberculosis

It is a type of tuberculosis that exclusively affects other organ rather lung parenchyma. It includes tuberculosis meningitis, abdominal tuberculosis, skeletal tuberculosis, Pott’s disease (spine TB), scrofula TB (lymphadenitis), and genitourinary (renal) tuberculosis.

### Data quality control measures

Joanna Briggs Institute (JBI’s) critical appraisal checklist for prevalence studies was used to assess quality of the included studies [24]. Studies with a score of 7 or more were included. The quality of each study was evaluated independently by two authors (M. S, and T. D) (**S4 File. JBI Critical Appraisal**). Discrepancies were solved by discussion with third independent reviewer (NG).

### Data extraction

The standardized data extraction tool of JBI MAStARI was used to extract data on Microsoft Excel 2016 spreadsheet. The name of the first author, publication year, region, sample size, frequency of delay and risk factors were extracted (Table 1). Two independent reviewers (MS and TD) extracted the data and cross-checked their consistency. We tried to contact the corresponding author(s) for further information, but we could not obtain a response from the corresponding author.

**Table 1:** Characteristics of the included studies on treatment seeking delay in Ethiopia, 2020.

S.No	Author	Year	Region	Study design	Sample size	Frequency
1.	Terefe G [17]	2018	SNNPR	cross sectional	398	230
2.	Melashu B [25]	2019	Amhara	cross sectional	170	102
3.	Tirusew M [18]	2019	Oromia	cross sectional	598	275
4.	Abdurahman S [26]	2018	Amhara	cross sectional	382	157
5.	Hailesillase [27]	2019	Tigray	cross sectional	422	132
6.	Mihret A [28]	2017	Amhara	cross sectional	605	240
7.	Kiros T [29]	2020	Tigray	cross sectional	875	392
8.	Tatek W [30]	2007	Oromia	cross sectional	198	73
9.	Yibeltal E [31]	2020	Amhara	cross sectional	300	126
10.	Getinet S [32]	2017	Addis Ababa	cross sectional	425	179
11.	Abdulbasit H [33]	2015	Oromia	cross sectional	362	133
12.	Mohammed Abdu [14]	2020	Oromia	cross sectional	387	211

### Statistical analysis

Statistical analyses were done by using Stata version 14.0 (Stata Corp. LP, College Station, United States of America). The heterogeneity test was conducted by using I-Squared (I^2^*)* statistics. The value of I^2^ statistics ranges from 0 to 100% and the 25, 50, and 75%, which represent low, medium, and high heterogeneity across the included studies, respectively [21]. The pooled prevalence of treatment seeking delay among TB patients was carried out using a random-effects (DerSimonian and Laird) method. To reduce the potential random variations between studies; the sources of heterogeneity were analyzed using the subgroup analysis, and meta-regression. Publication bias was checked subjectively by the funnel plots. Asymmetry of funnel plot was used as an indication of publication bias [34]. Egger’s weighted regression test at a p-value < 0.05 was used to objectively assess the presence of publication bias [35]. A leave-out-one sensitivity analysis was conducted by excluding each study at a time to identify the impact of each study on the overall estimate.

### Study selection

**Fig 1:**
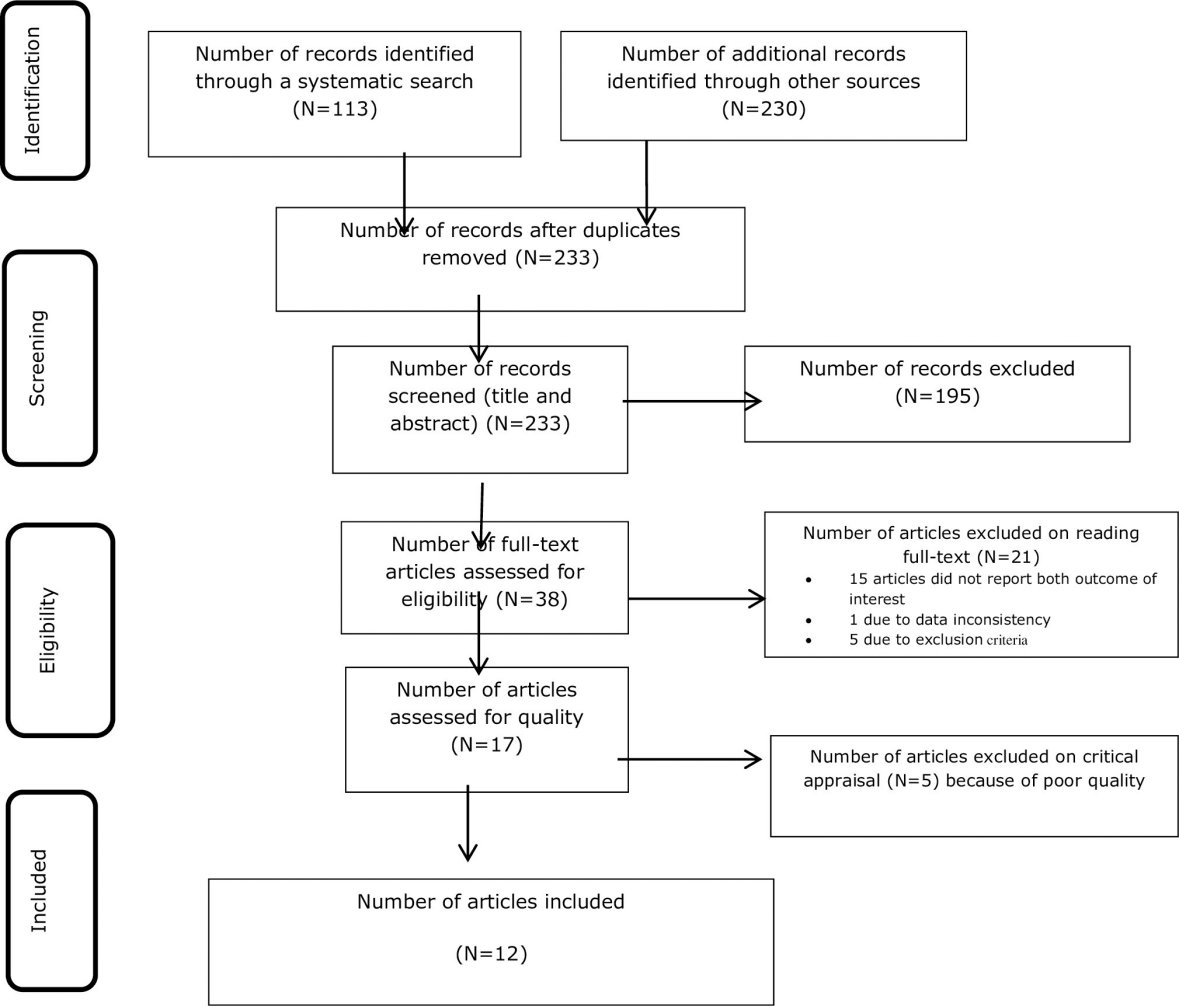
PRISMA flow diagram of treatment seeking delay among TB patients in Ethiopia, 2020.

In this systematic review and meta-analysis, out of total 343 articles retrieved, 113 articles were retrieved through electronic online and 230 articles were retrieved through manual searching. Among them, 110 duplicated records were removed and 195 articles were excluded through screening the title and abstracts. Then, the remaining 38 full articles were assessed for eligibility; among them, 15 articles did not report the outcome of interest, 1 article had data inconsistency, and 5 articles had not fulfilled other eligibility criteria. As a result, 21 articles were excluded through reading full texts based on a predefined inclusion, and/or exclusion criteria. After that, a total of 17 full-texts were assessed for a critical appraisal. Five studies failed to meet the inclusion criteria; hence, excluded due to poor methodological quality. Finally, only 12 published studies met the inclusion criteria, and thereby contributed to our systematic review and meta-analysis (Fig 1).

## Result

### Characteristics of the included studies

A total of 12 studies with 5122 patients were included in this meta analysis and systematic review. The total number of patients who delayed in seeking health care was 2250. All of the included studies were cross-sectional studies. The sample size ranged from 1700 [25] to 875 [29]. The studies were conducted between 2013 and 2020 across the country. Of these studies, 4 were from the Amhara region, 4 studies were from the Oromia region, and 2 studies were from the Tigray region, and 1 study from SNNPR **(**Southern Nations, Nationalities, and Peoples Region) and Addis Ababa city administration (Table 1).

### Prevalence of treatment seeking delay

**Fig 2:**
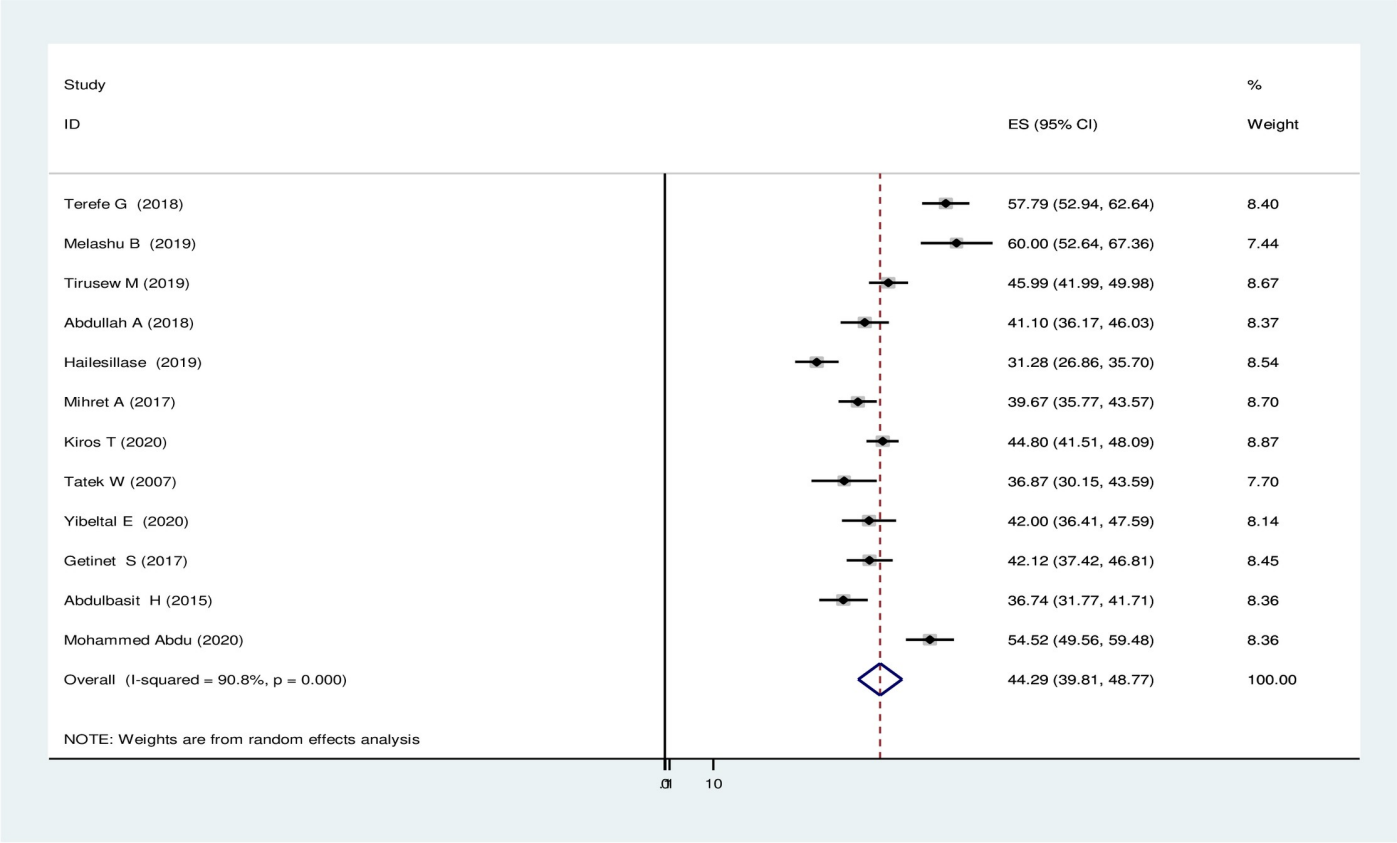
Pooled prevalence of treatment seeking delay in seeking health service delivery among tuberculosis patients in Ethiopia, 2020.

The patient was delayed if it took more than twenty one days from presentation of major TB clinical symptoms to arrival of formal health care institution. The pooled effect size of treatment seeking delay using the fixed effect model showed significant heterogeneity across the studies with (I^2^) of 90.8% (P ≤ 0.001). Therefore, we performed the analysis with a random-effect model with 95% CI to regulate for the observed variability. Accordingly, the national pooled prevalence of treatment seeking delay was 44.29% (95% CI: 39.805, 48.771) (Fig 2).

### Subgroup analysis

**Fig 3:**
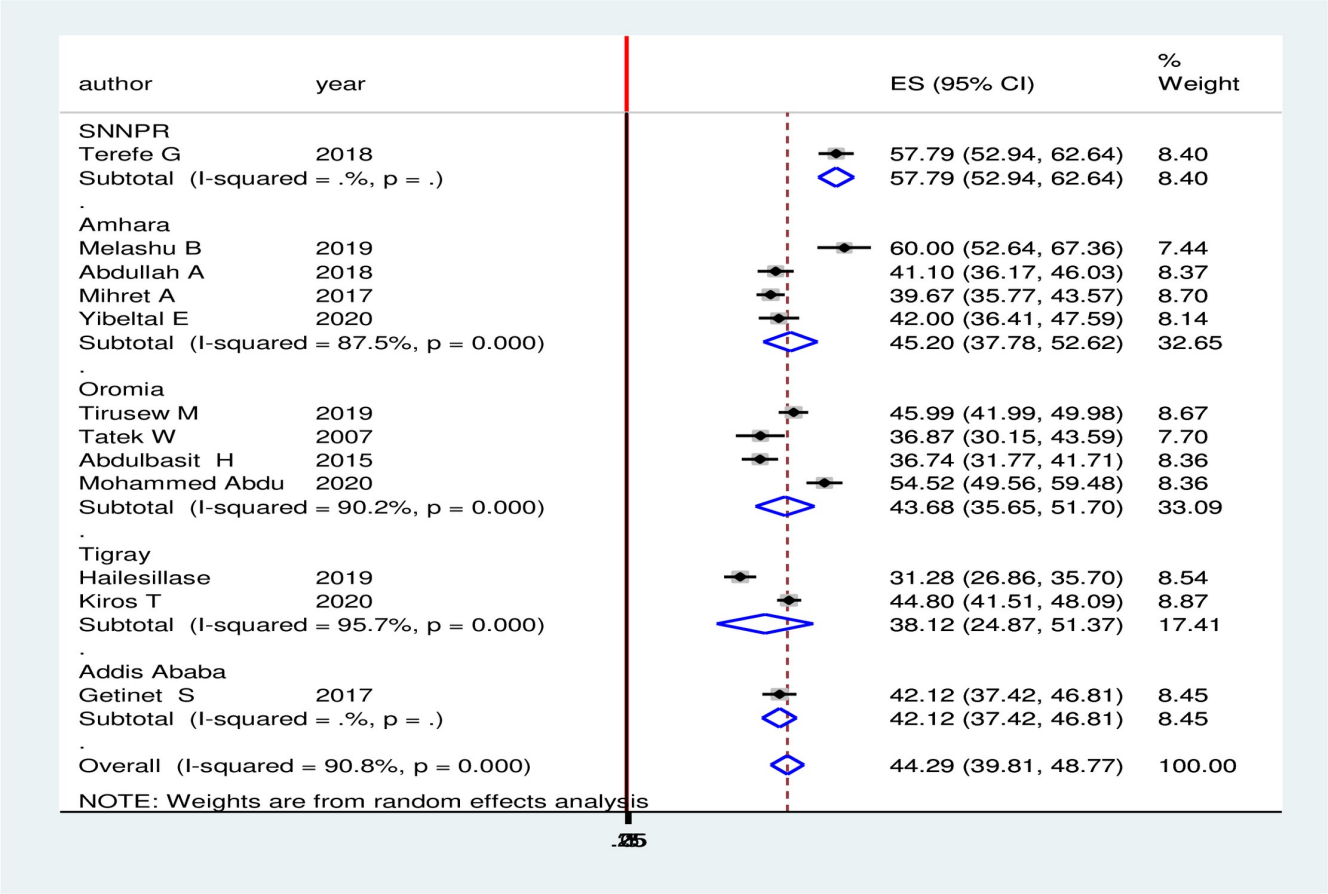
Subgroup analyses by region among TB patient in Ethiopia, 2020.

To adjust and minimize the reported heterogeneity of this study, we performed a subgroup analysis based on the region in Ethiopia. Accordingly, the highest rate of treatment seeking delay was observed in Oromia region 57.789% (95% CI: 52.937, 62.641) followed by Amhara region 45.20% (95% CI: 37.784, 52.620) and Oromia region 43.677% (95% CI: 35.653, 51.701). On the other hand, the lowest pooled prevalence of delay in health seeking was in Tigray region 38.124% (95% CI: -24.875, 51.372, and Addis Ababa city administration 42.118% (95% CI: 37.423, 46.812 (Fig 3).

### Heterogeneity and publication bias

**Fig 4:**
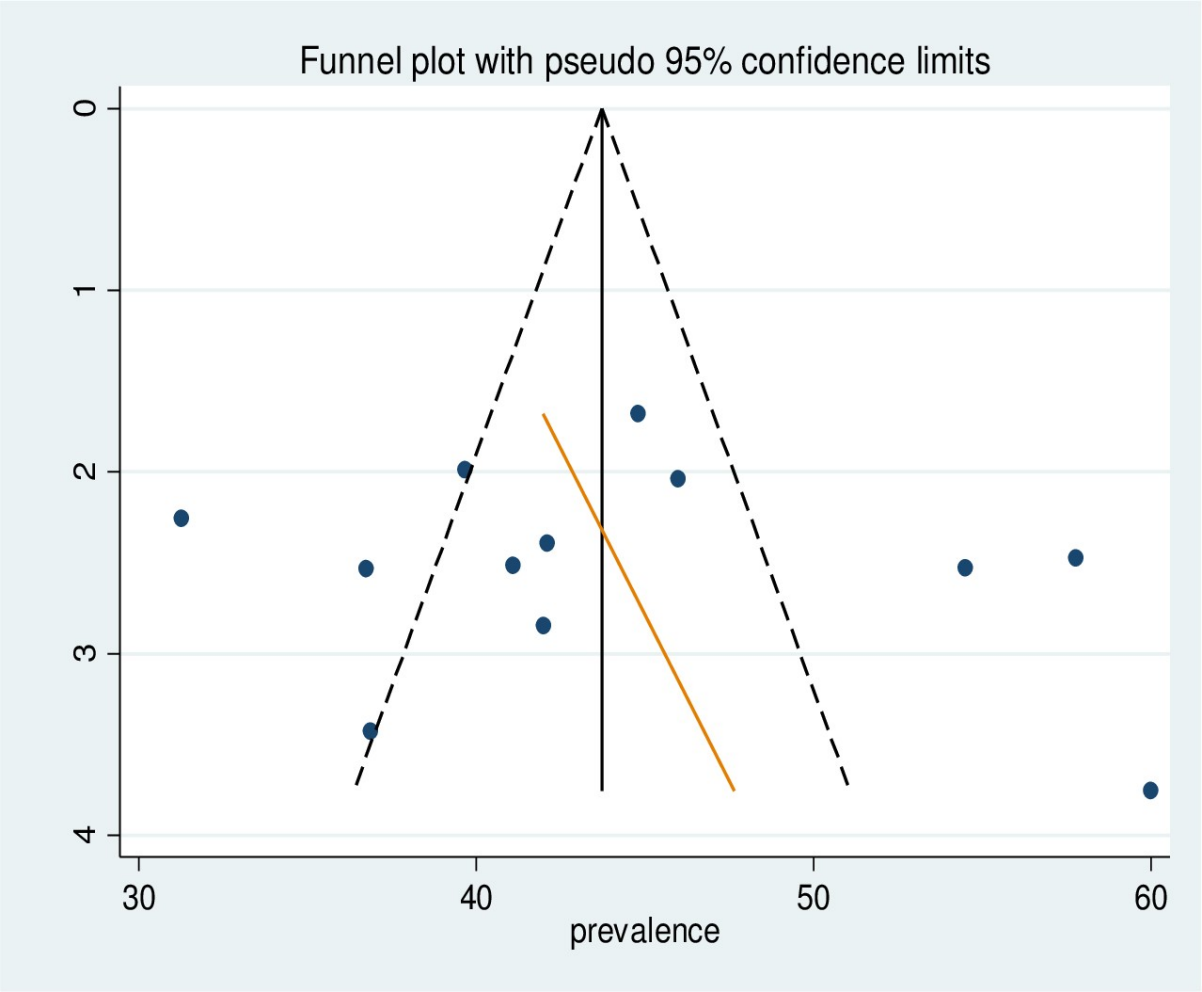
Funnel plot to test publication bias in 14 studies with 95% confidence limit.

Meta-regression was also conducted to identify the source of heterogeneity using sample size and year of publication as a covariate. It was indicated that there was no effect of sample size and year of publication on heterogeneity between studies (Table 2). The presence of publication bias was tested by Egger’s test and graphically by a funnel plot. Visual inspection of the funnel plot showed asymmetrical distribution, and the Egger test showed insignificant (P = 0.348) (Fig 4).

**Table 2:** Meta-regression analysis of factors affecting between-study heterogeneity.

Type of outcome	Heterogeneity source	Coefficients	Std. Err.	P>t
Delay to seek health care	Publication year	-.0143786	.0145204	0.348
	Sample size	1.239396	.7957167	0.154

### Sensitivity analysis

We also executed sensitivity analysis by removing studies step by step to evaluate the effect of a single study on the overall effect estimate. The results indicated that removing a single study did not have a significant influence on pooled effects (Table 3).

**Table 3:** Sensitivity analysis of pooled prevalence for each study being removed one at a time.

Study omitted	Estimate	[95% Conf. Interval]	
Terefe G (2018) [17]	43.015514	38.887733	47.143299
Melashu B (2019) [25]	43.028976	38.666386	47.391567
Tirusew M (2019) [18]	44.140415	39.171921	49.10891
Abdullah A (2018)	44.588055	39.719296	49.45681
Hailesillase (2019) [27]	45.48138	41.283703	49.679058
Mihret A (2017) [28]	44.736866	39.824715	49.649021
Kiros T (2020) [29]	44.257614	39.142715	49.372517
Tatek W (2007) [30]	44.909893	40.205029	49.614761
Yibeltal E (2020) [31]	44.498741	39.661049	49.33643
Getinet S (2017) [32]	44.498489	39.594158	49.402821
Abdulbasit H (2015) [33]	44.978291	40.257931	49.698647
Mohammed Abdu (2020) [14]	43.343212	38.868977	47.817444
Combined	44.288032	39.805081	48.770983

### Factors associated with treatment seeking delay in seeking health care

12 variables were extracted to identify factors associated with delay in seeking health care. Of these, 8 variables (age, Information about TB, residence, distance, social stigma, occupational status, marital status and educational status) had a strong significant association with delay in treatment seeking at P-value of less than 0.001(Table 4).

**Table 4:** Factors associated with delay in seeking health care among tuberculosis patients visited health facilities in Ethiopia.

Determinants (References)	Comparison	Number of Studies	Sample Size	OR (95% CI)	P value	I^2^ (%)	Heterogeneity test (P value)	Egger test (P value)
Information about TB [14,17,18,25,27,32]	Good Vs poor	6	2379	0.975(0.6391.48)	< 0.001	83.6	0.907	0.767
Residence [14,17,28,29,36]	Rural Vs Urban	5	2715	3.459(1.469–8.148)	< 0.001	95.8	0.005	0.939
Sex [17,28,29,36]	Male Vs Female	4	2370	0.972 (0.893–1.059)	0.015	71.4	0.517	0.482
Marital status [28,32,36]	Married Vs Others	3	1392	0.979 (0.454–2.112)	< 0.001	90.4	0.286	0.661
Distance [14,18,29]	≥10km Vs <10km	3	1858	1.631(0.858–3.101)	< 0.001	90.2	0.136	0.952
Social stigma [27,32]	High Vs low	2	847	1.187(0.293–4.798)	< 0.001	95.5	0.810	-
Age [17,27,28]	≥ 45 Vs <45	3	1422	0.363(0.082–1.598)	< 0.001	96.2	0.180	0.433
Occupation [17,27,28,32,36]	Employed Vs Others	5	2944	0.670(0.608–0.739)	< 0.001	93.6	0.001	0.586
HIV Sero status [36,37]	Positive Vs Negative	2	1237	0.502(0.257–0.981)	0.160	49	< 0.001	-
Other treatment sought [14,18,29]	Not Sought Vs sought	3	2212	7.192(5.587–9.257)	0.001	85	< 0.001	0.044
TB can be cured [17,18,25]	Yes Vs No	3	1155	0.586(0.321–1.070)	0.024	73.1	0.082	0.975
Type of TB [14,25,33]	ETPTB Vs PTB	3	911	2.520(1.761–3.605)	0.180	95.3	0.001	0.882
Educational level [14,17,28,38,39]	≤primary school Vs > primary school	5	2164	11.720(1.006–2.938)	<0.001	85.0	0.047	0.923

NB: Vs means versus.

Patients who had good information about tuberculosis were 2.5% less likely to delay in treatment seeking than the patients who had poor knowledge about tuberculosis (OR: 0.975 (95% CI 0.639–1.487), P <0.001, I^2^: 83.6%; the heterogeneity test (P = 0.907) showed significant evidence of variation across studies, and Egger’s test showed no evidence of publication bias (p = 0.767)).

Additionally, patients who had a perception of tuberculosis as a curable disease were about 41.4% less likely to delay than who were not(OR: 0.586 ((95% CI 0.321–1.070), P = 0.024, I^2^: 66.8%), the heterogeneity test (p = 0.082) showed significant variation across studies, and Egger’s test showed no evidence of publication bias (P = 0.975)).

Patients who did not seek health care providers on a first contact had about 7 times more likely to delay in treatment seeking than patients who sought formal health care provider on a first contact (OR: 7.192 ((95% CI 5.587–9.257), p = 0.001, I^2^: 85%), the heterogeneity test (P < 0.001) showed no significant variation across studies, and Egger’s test showed there is evidence of publication bias (P = 0.044)).

Furthermore, patients from rural areas had about four times more likely to have a treatment seeking delay than the patients who visited health facility from urban (OR: 3.459((95% CI 1.469–8.148), p < 0.001, I^2^: 89%), the heterogeneity test (P = 0.005) showed no significant variation across studies, and the result of Egger’s test showed no evidence of publication bias (P = 0.939)). Those who travelled more than 10km to reach health facility were almost two times more likely to delay seeking health care than who travelled less than 10km (OR: 1.631((95% CI (10.858–3.101), P = 0.001, I^2^: 90.2%). The heterogeneity test (P = 0.136) showed significant variation across studies, and Egger’s test showed no evidence of publication bias (P = 0.952)).The others independent predictors of delay was extra pulmonary TB (OR: 2.520 ((95% CI 1.761–3.605), 0.180), and lower educational level (OR 11.720 ((95% CI 1.006–2.938), P <0.001).

## Discussion

This review revealed a substantial delay in treatment seeking behaviors among TB patients in Ethiopia. It was found that the national pooled prevalence of treatment seeking delay was 44.29%. This high prevalence greatly affects tuberculosis control and prevention program. On the other hand, the prevalence of treatment seeking delay was shorter than previous studies in Ethiopia [17,28,31] and higher than other reports from Ethiopia [30,40,41]. This might be partly due to lack of awareness and traditional medicinal practices across different region of Ethiopia [28].

According to the findings of this review, patients who did not visit formal health care provider on a first contact were a significant risk factor of treatment seeking delay which is consistent with the findings of other similar studies [15,42,43]. Another study also found that the use of informal treatment provider was a strong predictor of treatment seeking delay [41]. This study also in line with other previous studies in Ethiopia [14,15], and Nigeria [44]. This might be due to low confidence in treatment in a health facility and high cultural beliefs of patients for treatment of TB. Besides, the use of some home remedies or antibiotics might reduce or mask the symptoms of the illness which might hinder the timely presentation of the patient to the health care facility [29].

This meta analysis showed that patients from rural areas was a risk factor of treatment seeking delay which agrees with other similar studies [45,46]. In contrary to the current review, another study found that the remoteness of their residence was not found to be associated with delay [17]. In line with current review, other studies conducted in Ethiopia found that rural residents had higher odds of delay compared to the urban residents [26,47]. This might be related to the poor access of rural residents to health information and health facility compared to the urban residents. In addition, the low delay among urban residents could also be as a result of regular and intensive health education offered by health care providers about the disease and easy accessibly to health care institutions.

This review found that long walking distance between health facilities and patient home was a risk factor of delay. A patient who travelled farther than 10 kilometers from health facilities, have a higher delay as compared to those who travelled less than 10 kilometers. The result of this study is in line with other studies conducted in Oromia Special Zone and North Wollo [14,18]. This might be due to rural residency makes it difficult to travel to health facility due to lack of road or distance [48]. Unlike this study, the distance to health facility had not found to affect the treatment seeking delay [11,49,50]. This difference might be due to the fact that the majority of the respondents of those studies live in a distance of less than 2 hours travel. In contrast to the current review, the other studies conducted in Tanzania and Chad also showed shorter time delay by rural patient. This differences could be related with accessibility to the health facility as about 87.5% and 89% of the study participants in Tanzania and Chad had lived with less than 10km from the health facility respectively [12,51].

According to the finding of this review, patients with extra pulmonary TB patients had a higher risk of treatment seeking delay than its counterparts which agrees with other similar study [14]. Similarly, another study also found that extra pulmonary Tuberculosis is significantly associated with delay [36,40]. This could be due to the fact that patient who had extra pulmonary tuberculosis is less likely to produce the cough and expectorate sputum that helped the patients to seek health facility.

The present review showed that treatment seeking delay has been associated with various Sociodemographic factors which agrees with other multiple studies conducted in Ethiopia and elsewhere [30,52–54]. In this review, unemployed patients took relatively long time to go to health care facility compared to employed patients. Similarly, another study found that unemployed patients were independent predictors of treatment seeking delay [55]. This finding is in agreement with study findings done in Uganda, where farmers were experiencing unacceptable patient delay compared to the rest of the respondents [56]. This could be due to lack of awareness on tuberculosis among farmers or unemployed patients, which eventually affects health care seeking behavior.

The present study revealed that age was strongly associated with treatment seeking delay. In contrast to this study, another study found that age was not statistically significant with treatment seeking delay [54]. However, a study conducted in Hadiya Zone, southern Ethiopia showed younger patients aged less than 19 years and elderly patients aged more than 45 years took a longer time to go to the health facility in seeking treatment for their symptoms [55]. This may be due to younger and elderly patients are economically dependent on others in Ethiopia.

The findings of this study also revealed that level of information about tuberculosis and educational status were strongly associated with treatment seeking delay. Other studies conducted in Ethiopia reported lower educational level as an independent predictor of treatment seeking delay [29,47]. Patients who had poor information about TB were more likely to have longer patients delay than those who had good information about TB [57]. In addition, the absence of formal education was strongly associated with treatment seeking delay [47]. This might be due the fact that patient’s who attended higher education levels might have better information about TB to early seek treatment [58].

According to the finding of this review, patient perception on TB was significantly associated with a treatment seeking delay which is consistent with a study conducted on an analysis of symptom duration among Ethiopian pulmonary tuberculosis patients [59]. Similarly, another study done in southern Ethiopia showed that patients who thought TB can be cured was more likely to delay than its counterpart. In this case, the majority of patients believed that their illness will disappear by itself; hence, patients may not visit health facilities [60]. In contrast to our study, the finding of another study done in Uganda revealed that patients who thought TB as a curable disease were more likely to delay seeking treatment than its counterpart [61]. This may be because of their awareness that makes them reluctant.

Being HIV Sero positive patients and being affected with high social stigma are other factors associated with treatment seeking delay which agrees with another study [39]. Similarly, a study conducted on health care seeking delay among pulmonary tuberculosis patients in the North West zone of Tigrai region, North Ethiopia showed that the presence of stigma was independent predictors of treatment seeking delay [27]. The linking of TB with HIV progressively created attitudes and perceptions that have become a source of stigma associated with TB as well as fears of being infected with HIV, and has been cited in several studies as one of the reasons for delays in treatment seeking [62,63].

The limitations of this review were some studies did not contain sufficient predictor variables. Besides, other studies categories predictor variables like patients’ income differently. As a result, we excluded predictor variables like patients’ income from the data analysis.

## Conclusion

In this meta analysis and systematic review, we identified a substantial treatment seeking delay among TB patients in Ethiopia. However, the treatment seeking delay varies among different studies and regions of the country. Furthermore, delay was affected by age, information about TB, residence, distance, social stigma, occupational status, marital status and educational status. And, the independent predictors of delay were treatment sought before formal health care provider, residence of the patient, type of TB, educational level, and distance from a health facility. Thus, we recommend health extension workers, health professionals and other stakeholders to focus on patient education, and to continuously mobilize the whole communities on early treatment seeking with a special emphasis given to where treatment sought before formal health care provider, rural resident, extra pulmonary TB, and a patient living farther than 10km distance from health facility.

## Supporting information

S1 File Search details. This is the search details of treatment seeking delay. (DOCX)

S2 File Prisma 2017 checklist. This is the 2017 Prisma checklist. (DOC)

S3 File This is the data of treatment seeking delay. (XLSX)

S4 File JBI Critical Appraisal. This is the JBI Critical Appraisal of treatment seeking delay.
(DOCX)

## Abbreviations

CI: Confidence Interval
ETPTB: Extra Pulmonary Tuberculosis
HCP: Health Care Provider
OR: Odds Ratio
PRISMA: Preferred Reporting Items for Systematic Reviews and Meta-Analyses
PTB: Pulmonary Tuberculosis
Vs: Versus
