# Supplementary material for: Treatment seeking delay and associated factors among tuberculosis patients attending health facility in Ethiopia from 2000 to 2020: A systematic review and meta analysis
Source: PLoS One. 2021 Jul 1;16(7):e0253746. doi: 10.1371/journal.pone.0253746 (PMC8248725; doi:10.1371/journal.pone.0253746)
Supplement: S1 File — (DOCX) [file pone.0253746.s001.docx]

**Search strategy**

The search aimed to find both published and unpublished studies. The search was restricted to studies published in the English language before October 24, 2020, G. C. A three-step search strategy was utilized in this review. An initial limited search of PubMed, Cochrane Database, Cinahl, Scopus, Mednar, and Google Scholar was undertaken, followed by analysis of the text words contained in the title and abstract and of the index terms used to describe the article. A second search using all identified keywords and index terms was then undertaken across all included databases. Third, the reference lists of all identified reports and articles were searched for additional studies. The core search terms and phrases were “Tuberculosis”, “patient delay” "Time-to-Treatment", “diagnosis”, “treatment”, and “Ethiopia”.

The following terms with MeSH (Medical Subject Heading) Boolean operators were used to search PubMed

("Tuberculosis"[Text Word] OR "Tuberculosis"[Text Word] OR "TB"[Text Word] OR ("Tuberculosis"[MeSH Terms] OR "Tuberculosis"[Text Word])) AND ("tb diagnosis"[Text Word] OR "diagnosis"[Text Word] OR ("therapeutics"[MeSH Terms] OR "therapeutics"[All Fields] OR "treatments"[All Fields] OR "therapy"[MeSH Subheading] OR "therapy"[All Fields] OR "treatment"[All Fields] OR "treatment s"[All Fields]) OR "tb treatment"[Text Word] OR "tuberculosis diagnosis"[Text Word] OR "tuberculosis treatment"[Text Word] OR ((("diagnosis"[MeSH Subheading] OR "diagnosis"[MeSH Terms] OR "diagnosis"[Text Word]) AND "therapy"[MeSH Subheading]) OR "therapeutics"[MeSH Terms] OR "treatment"[Text Word])) AND ("Delay"[Text Word] OR "late"[Text Word] OR "Time-to-Treatment"[Text Word] OR ("Time-to-Treatment"[MeSH Terms] OR ("Delay"[All Fields] OR "delayed"[All Fields] OR "delaying"[All Fields] OR "delays"[All Fields]))) AND ("Ethiopia"[Text Word] OR ("Ethiopia"[MeSH Terms] OR "Ethiopia"[All Fields] OR "ethiopia s"[All Fields]) OR ("Ethiopia"[MeSH Terms] OR "Ethiopia"[Text Word]))
