# Supplementary material for: Treatment seeking delay and associated factors among tuberculosis patients attending health facility in Ethiopia from 2000 to 2020: A systematic review and meta analysis
Source: PLoS One. 2021 Jul 1;16(7):e0253746. doi: 10.1371/journal.pone.0253746 (PMC8248725; doi:10.1371/journal.pone.0253746)
Supplement: S4 File — This is the JBI Critical Appraisal of treatment seeking delay. (DOCX) [file pone.0253746.s004.docx]

**JBI Critical Appraisal Checklist for Studies Reporting Prevalence Data**

Reviewer M.S√ Date October 24, 2020, G. C

Author Terefe Gone Year 2018 Record Number 01

|  | Yes | No | Unclear | Not applicable |
| --- | --- | --- | --- | --- |
| 1. Was the sample frame appropriate to address the target population? | □√ | □ | □ | □ |
| 1. Were study participants sampled in an appropriate way? | □√ | □ | □ | □ |
| 1. Was the sample size adequate? | □ | □X | □ | □ |
| 1. Were the study subjects and the setting described in detail? | □√ | □ | □ | □ |
| 1. Was the data analysis conducted with sufficient coverage of the identified sample? | □√ | □ | □ | □ |
| 1. Were valid methods used for the identification of the condition? | □√ | □ | □ | □ |
| 1. Was the condition measured in a standard, reliable way for all participants? | □√ | □ | □ | □ |
| 1. Was there appropriate statistical analysis? | □√ | □ | □ | □ |
| 1. Was the response rate adequate, and if not, was the low response rate managed appropriately? | □√ | □ | □ | □ |

Overall appraisal: Include √□ Exclude □ Seek further info □

Comments (Including reason for exclusion

**JBI Critical Appraisal Checklist for Studies Reporting Prevalence Data**

Reviewer T.D Date October 24, 2020, G. C

Author Terefe Gone Year 2018 Record Number 01

|  | Yes | No | Unclear | Not applicable |
| --- | --- | --- | --- | --- |
| 1. Was the sample frame appropriate to address the target population? | □√ | □ | □ | □ |
| 1. Were study participants sampled in an appropriate way? | □√ | □ | □ | □ |
| 1. Was the sample size adequate? | □ | □X | □ | □ |
| 1. Were the study subjects and the setting described in detail? | □√ | □ | □ | □ |
| 1. Was the data analysis conducted with sufficient coverage of the identified sample? | □√ | □ | □ | □ |
| 1. Were valid methods used for the identification of the condition? | □√ | □ | □ | □ |
| 1. Was the condition measured in a standard, reliable way for all participants? | □√ | □ | □ | □ |
| 1. Was there appropriate statistical analysis? | □√ | □ | □ | □ |
| 1. Was the response rate adequate, and if not, was the low response rate managed appropriately? | □√ | □ | □ | □ |

Overall appraisal: Include √ □ Exclude □ Seek further info □

Comments (Including reason for exclusion)

**JBI Critical Appraisal Checklist for Studies Reporting Prevalence Data**

Reviewer M.S Date October 24, 2020, G. C

Author [Melashu Balew](https://www.ncbi.nlm.nih.gov/pubmed/?term=Shiferaw%20MB%5BAuthor%5D&cauthor=true&cauthor_uid=30991995) Year 2019 Record Number 02

|  | Yes | No | Unclear | Not applicable |
| --- | --- | --- | --- | --- |
| 1. Was the sample frame appropriate to address the target population? | □√ | □ | □ | □ |
| 1. Were study participants sampled in an appropriate way? | □√ | □ | □ | □ |
| 1. Was the sample size adequate? | □ | □X | □ | □ |
| 1. Were the study subjects and the setting described in detail? | □√ | □ | □ | □ |
| 1. Was the data analysis conducted with sufficient coverage of the identified sample? | □√ | □ | □ | □ |
| 1. Were valid methods used for the identification of the condition? | □√ | □ | □ | □ |
| 1. Was the condition measured in a standard, reliable way for all participants? | □√ | □ | □ | □ |
| 1. Was there appropriate statistical analysis? | □√ | □ | □ | □ |
| 1. Was the response rate adequate, and if not, was the low response rate managed appropriately? | □√ | □ | □ | □ |

Overall appraisal: Include √ □ Exclude □ Seek further info □

Comments (Including reason for exclusion)

**JBI Critical Appraisal Checklist for Studies Reporting Prevalence Data**

Reviewer T.D Date October 24, 2020, G. C

Author [Melashu Balew](https://www.ncbi.nlm.nih.gov/pubmed/?term=Shiferaw%20MB%5BAuthor%5D&cauthor=true&cauthor_uid=30991995) Year 2019 Record Number 02

|  | Yes | No | Unclear | Not applicable |
| --- | --- | --- | --- | --- |
| 1. Was the sample frame appropriate to address the target population? | □√ | □ | □ | □ |
| 1. Were study participants sampled in an appropriate way? | □√ | □ | □ | □ |
| 1. Was the sample size adequate? | □ | □X | □ | □ |
| 1. Were the study subjects and the setting described in detail? | □√ | □ | □ | □ |
| 1. Was the data analysis conducted with sufficient coverage of the identified sample? | □√ | □ | □ | □ |
| 1. Were valid methods used for the identification of the condition? | □√ | □ | □ | □ |
| 1. Was the condition measured in a standard, reliable way for all participants? | □√ | □ | □ | □ |
| 1. Was there appropriate statistical analysis? | □√ | □ | □ | □ |
| 1. Was the response rate adequate, and if not, was the low response rate managed appropriately? | □√ | □ | □ | □ |

Overall appraisal: Include √ □ Exclude □ Seek further info □

Comments (Including reason for exclusion)

**JBI Critical Appraisal Checklist for Studies Reporting Prevalence Data**

Reviewer M.S Date October 24, 2020, G. C

# Author [Tirusew Maru](https://www.ncbi.nlm.nih.gov/pubmed/?term=Wondawek%20TM%5BAuthor%5D&cauthor=true&cauthor_uid=31727034)Year 2019 Record Number 03

|  | Yes | No | Unclear | Not applicable |
| --- | --- | --- | --- | --- |
| 1. Was the sample frame appropriate to address the target population? | □√ | □ | □ | □ |
| 1. Were study participants sampled in an appropriate way? | □√ | □ | □ | □ |
| 1. Was the sample size adequate? | □√ | □ | □ | □ |
| 1. Were the study subjects and the setting described in detail? | □√ | □ | □ | □ |
| 1. Was the data analysis conducted with sufficient coverage of the identified sample? | □√ | □ | □ | □ |
| 1. Were valid methods used for the identification of the condition? | □√ | □ | □ | □ |
| 1. Was the condition measured in a standard, reliable way for all participants? | □√ | □ | □ | □ |
| 1. Was there appropriate statistical analysis? | □√ | □ | □ | □ |
| 1. Was the response rate adequate, and if not, was the low response rate managed appropriately? | □√ | □ | □ | □ |

Overall appraisal: Include √ □ Exclude □ Seek further info □

Comments (Including reason for exclusion)

**JBI Critical Appraisal Checklist for Studies Reporting Prevalence Data**

Reviewer T.D Date October 24, 2020, G. C

# Author [Tirusew Maru](https://www.ncbi.nlm.nih.gov/pubmed/?term=Wondawek%20TM%5BAuthor%5D&cauthor=true&cauthor_uid=31727034)Year 2019 Record Number 03

|  | Yes | No | Unclear | Not applicable |
| --- | --- | --- | --- | --- |
| 1. Was the sample frame appropriate to address the target population? | □√ | □ | □ | □ |
| 1. Were study participants sampled in an appropriate way? | □√ | □ | □ | □ |
| 1. Was the sample size adequate? | □√ | □ | □ | □ |
| 1. Were the study subjects and the setting described in detail? | □√ | □ | □ | □ |
| 1. Was the data analysis conducted with sufficient coverage of the identified sample? | □√ | □ | □ | □ |
| 1. Were valid methods used for the identification of the condition? | □√ | □ | □ | □ |
| 1. Was the condition measured in a standard, reliable way for all participants? | □√ | □ | □ | □ |
| 1. Was there appropriate statistical analysis? | □√ | □ | □ | □ |
| 1. Was the response rate adequate, and if not, was the low response rate managed appropriately? | □√ | □ | □ | □ |

Overall appraisal: Include √ □ Exclude □ Seek further info □

Comments (Including reason for exclusion)

**JBI Critical Appraisal Checklist for Studies Reporting Prevalence Data**

Reviewer M.S Date October 24, 2020, G. C

# Author Abdurahman Seid Year 2018 Record Number 04

|  | Yes | No | Unclear | Not applicable |
| --- | --- | --- | --- | --- |
| 1. Was the sample frame appropriate to address the target population? | □√ | □ | □ | □ |
| 1. Were study participants sampled in an appropriate way? | □√ | □ | □ | □ |
| 1. Was the sample size adequate? | □ | □X | □ | □ |
| 1. Were the study subjects and the setting described in detail? | □√ | □ | □ | □ |
| 1. Was the data analysis conducted with sufficient coverage of the identified sample? | □√ | □ | □ | □ |
| 1. Were valid methods used for the identification of the condition? | □√ | □ | □ | □ |
| 1. Was the condition measured in a standard, reliable way for all participants? | □√ | □ | □ | □ |
| 1. Was there appropriate statistical analysis? | □√ | □ | □ | □ |
| 1. Was the response rate adequate, and if not, was the low response rate managed appropriately? | □√ | □ | □ | □ |

Overall appraisal: Include √ □ Exclude □ Seek further info □

Comments (Including reason for exclusion)

**JBI Critical Appraisal Checklist for Studies Reporting Prevalence Data**

Reviewer T.D Date October 24, 2020, G. C

# Author Abdurahman Seid Year 2018 Record Number 04

|  | Yes | No | Unclear | Not applicable |
| --- | --- | --- | --- | --- |
| 1. Was the sample frame appropriate to address the target population? | □√ | □ | □ | □ |
| 1. Were study participants sampled in an appropriate way? | □√ | □ | □ | □ |
| 1. Was the sample size adequate? | □ | □X | □ | □ |
| 1. Were the study subjects and the setting described in detail? | □√ | □ | □ | □ |
| 1. Was the data analysis conducted with sufficient coverage of the identified sample? | □√ | □ | □ | □ |
| 1. Were valid methods used for the identification of the condition? | □√ | □ | □ | □ |
| 1. Was the condition measured in a standard, reliable way for all participants? | □√ | □ | □ | □ |
| 1. Was there appropriate statistical analysis? | □√ | □ | □ | □ |
| 1. Was the response rate adequate, and if not, was the low response rate managed appropriately? | □ | □X | □ | □ |

Overall appraisal: Include √ □ Exclude □ Seek further info □

Comments (Including reason for exclusion)

**JBI Critical Appraisal Checklist for Studies Reporting Prevalence Data**

Reviewer M.S Date October 24, 2020, G. C

# Author Hailesillase Year 2019 Record Number 05

|  | Yes | No | Unclear | Not applicable |
| --- | --- | --- | --- | --- |
| 1. Was the sample frame appropriate to address the target population? | □√ | □ | □ | □ |
| 1. Were study participants sampled in an appropriate way? | □√ | □ | □ | □ |
| 1. Was the sample size adequate? | □√ | □ | □ | □ |
| 1. Were the study subjects and the setting described in detail? | □√ | □ | □ | □ |
| 1. Was the data analysis conducted with sufficient coverage of the identified sample? | □ | □X | □ | □ |
| 1. Were valid methods used for the identification of the condition? | □√ | □ | □ | □ |
| 1. Was the condition measured in a standard, reliable way for all participants? | □√ | □ | □ | □ |
| 1. Was there appropriate statistical analysis? | □√ | □ | □ | □ |
| 1. Was the response rate adequate, and if not, was the low response rate managed appropriately? | □√ | □ | □ | □ |

Overall appraisal: Include √ □ Exclude □ Seek further info □

Comments (Including reason for exclusion)

Reviewer T.D Date October 24, 2020, G. C

# Author Hailesillase Year 2019 Record Number 05

|  | Yes | No | Unclear | Not applicable |
| --- | --- | --- | --- | --- |
| 1. Was the sample frame appropriate to address the target population? | □√ | □ | □ | □ |
| 1. Were study participants sampled in an appropriate way? | □√ | □ | □ | □ |
| 1. Was the sample size adequate? | □√ | □ | □ | □ |
| 1. Were the study subjects and the setting described in detail? | □√ | □ | □ | □ |
| 1. Was the data analysis conducted with sufficient coverage of the identified sample? | □√ | □ | □ | □ |
| 1. Were valid methods used for the identification of the condition? | □√ | □ | □ | □ |
| 1. Was the condition measured in a standard, reliable way for all participants? | □√ | □ | □ | □ |
| 1. Was there appropriate statistical analysis? | □√ | □ | □ | □ |
| 1. Was the response rate adequate, and if not, was the low response rate managed appropriately? | □√ | □ | □ | □ |

Overall appraisal: Include √ □ Exclude □ Seek further info □

Comments (Including reason for exclusion)

**JBI Critical Appraisal Checklist for Studies Reporting Prevalence Data**

Reviewer M.S Date October 24, 2020, G. C

# Author [Mihret Asres](https://pubmed.ncbi.nlm.nih.gov/?term=Asres+M&cauthor_id=28500803)  Year 2017 Record Number 06

|  | Yes | No | Unclear | Not applicable |
| --- | --- | --- | --- | --- |
| 1. Was the sample frame appropriate to address the target population? | □√ | □ | □ | □ |
| 1. Were study participants sampled in an appropriate way? | □√ | □ | □ | □ |
| 1. Was the sample size adequate? | □√ | □ | □ | □ |
| 1. Were the study subjects and the setting described in detail? | □√ | □ | □ | □ |
| 1. Was the data analysis conducted with sufficient coverage of the identified sample? | □√ | □ | □ | □ |
| 1. Were valid methods used for the identification of the condition? | □√ | □ | □ | □ |
| 1. Was the condition measured in a standard, reliable way for all participants? | □√ | □ | □ | □ |
| 1. Was there appropriate statistical analysis? | □√ | □ | □ | □ |
| 1. Was the response rate adequate, and if not, was the low response rate managed appropriately? | □√ | □ | □ | □ |

Overall appraisal: Include √ □ Exclude □ Seek further info □

Comments (Including reason for exclusion)

**JBI Critical Appraisal Checklist for Studies Reporting Prevalence Data**

Reviewer T.D Date October 24, 2020, G. C

# Author [Mihret Asres](https://pubmed.ncbi.nlm.nih.gov/?term=Asres+M&cauthor_id=28500803)  Year 2017 Record Number 06

|  | Yes | No | Unclear | Not applicable |
| --- | --- | --- | --- | --- |
| 1. Was the sample frame appropriate to address the target population? | □√ | □ | □ | □ |
| 1. Were study participants sampled in an appropriate way? | □√ | □ | □ | □ |
| 1. Was the sample size adequate? | □√ | □ | □ | □ |
| 1. Were the study subjects and the setting described in detail? | □√ | □ | □ | □ |
| 1. Was the data analysis conducted with sufficient coverage of the identified sample? | □√ | □ | □ | □ |
| 1. Were valid methods used for the identification of the condition? | □√ | □ | □ | □ |
| 1. Was the condition measured in a standard, reliable way for all participants? | □√ | □ | □ | □ |
| 1. Was there appropriate statistical analysis? | □√ | □ | □ | □ |
| 1. Was the response rate adequate, and if not, was the low response rate managed appropriately? | □√ | □ | □ | □ |

Overall appraisal: Include √ □ Exclude □ Seek further info □

Comments (Including reason for exclusion)

**JBI Critical Appraisal Checklist for Studies Reporting Prevalence Data**

Reviewer M.S Date October 24, 2020, G. C

# Author [Kiros Tedla](https://pubmed.ncbi.nlm.nih.gov/?term=Tedla+K&cauthor_id=32822368)Year 2020 Record Number 07

|  | Yes | No | Unclear | Not applicable |
| --- | --- | --- | --- | --- |
| 1. Was the sample frame appropriate to address the target population? | □√ | □ | □ | □ |
| 1. Were study participants sampled in an appropriate way? | □√ | □ | □ | □ |
| 1. Was the sample size adequate? | □√ | □ | □ | □ |
| 1. Were the study subjects and the setting described in detail? | □√ | □ | □ | □ |
| 1. Was the data analysis conducted with sufficient coverage of the identified sample? | □√ | □ | □ | □ |
| 1. Were valid methods used for the identification of the condition? | □√ | □ | □ | □ |
| 1. Was the condition measured in a standard, reliable way for all participants? | □√ | □ | □ | □ |
| 1. Was there appropriate statistical analysis? | □√ | □ | □ | □ |
| 1. Was the response rate adequate, and if not, was the low response rate managed appropriately? | □√ | □ | □ | □ |

Overall appraisal: Include √ □ Exclude □ Seek further info □

Comments (Including reason for exclusion)

**JBI Critical Appraisal Checklist for Studies Reporting Prevalence Data**

Reviewer T.D Date October 24, 2020, G. C

# Author [Kiros Tedla](https://pubmed.ncbi.nlm.nih.gov/?term=Tedla+K&cauthor_id=32822368)Year 2020 Record Number 07

|  | Yes | No | Unclear | Not applicable |
| --- | --- | --- | --- | --- |
| 1. Was the sample frame appropriate to address the target population? | □√ | □ | □ | □ |
| 1. Were study participants sampled in an appropriate way? | □√ | □ | □ | □ |
| 1. Was the sample size adequate? | □√ | □ | □ | □ |
| 1. Were the study subjects and the setting described in detail? | □√ | □ | □ | □ |
| 1. Was the data analysis conducted with sufficient coverage of the identified sample? | □√ | □ | □ | □ |
| 1. Were valid methods used for the identification of the condition? | □√ | □ | □ | □ |
| 1. Was the condition measured in a standard, reliable way for all participants? | □√ | □ | □ | □ |
| 1. Was there appropriate statistical analysis? | □√ | □ | □ | □ |
| 1. Was the response rate adequate, and if not, was the low response rate managed appropriately? | □√ | □ | □ | □ |

Overall appraisal: Include √ □ Exclude □ Seek further info □

Comments (Including reason for exclusion)

**JBI Critical Appraisal Checklist for Studies Reporting Prevalence Data**

Reviewer M.S Date October 24, 2020, G. C

# Author Tatek Wondimu Year 2007 Record Number 08

|  | Yes | No | Unclear | Not applicable |
| --- | --- | --- | --- | --- |
| 1. Was the sample frame appropriate to address the target population? | □√ | □ | □ | □ |
| 1. Were study participants sampled in an appropriate way? | □√ | □ | □ | □ |
| 1. Was the sample size adequate? | □ | □X | □ | □ |
| 1. Were the study subjects and the setting described in detail? | □ | □X | □ | □ |
| 1. Was the data analysis conducted with sufficient coverage of the identified sample? | □√ | □ | □ | □ |
| 1. Were valid methods used for the identification of the condition? | □√ | □ | □ | □ |
| 1. Was the condition measured in a standard, reliable way for all participants? | □√ | □ | □ | □ |
| 1. Was there appropriate statistical analysis? | □√ | □ | □ | □ |
| 1. Was the response rate adequate, and if not, was the low response rate managed appropriately? | □√ | □ | □ | □ |

Overall appraisal: Include √ □ Exclude □ Seek further info □

Comments (Including reason for exclusion)

**JBI Critical Appraisal Checklist for Studies Reporting Prevalence Data**

Reviewer T.D Date October 24, 2020, G. C

# Author Tatek Wondimu Year 2007 Record Number 08

|  | Yes | No | Unclear | Not applicable |
| --- | --- | --- | --- | --- |
| 1. Was the sample frame appropriate to address the target population? | □√ | □ | □ | □ |
| 1. Were study participants sampled in an appropriate way? | □√ | □ | □ | □ |
| 1. Was the sample size adequate? | □ | □X | □ | □ |
| 1. Were the study subjects and the setting described in detail? | □ | □X | □ | □ |
| 1. Was the data analysis conducted with sufficient coverage of the identified sample? | □√ | □ | □ | □ |
| 1. Were valid methods used for the identification of the condition? | □√ | □ | □ | □ |
| 1. Was the condition measured in a standard, reliable way for all participants? | □√ | □ | □ | □ |
| 1. Was there appropriate statistical analysis? | □√ | □ | □ | □ |
| 1. Was the response rate adequate, and if not, was the low response rate managed appropriately? | □√ | □ | □ | □ |

Overall appraisal: Include √ □ Exclude □ Seek further info □

Comments (Including reason for exclusion)

**JBI Critical Appraisal Checklist for Studies Reporting Prevalence Data**

Reviewer M.S Date October 24, 2020, G. C

# Author Yibeltal Estemech Year 2020 Record Number 09

|  | Yes | No | Unclear | Not applicable |
| --- | --- | --- | --- | --- |
| 1. Was the sample frame appropriate to address the target population? | □√ | □ | □ | □ |
| 1. Were study participants sampled in an appropriate way? | □√ | □ | □ | □ |
| 1. Was the sample size adequate? | □ | □X | □ | □ |
| 1. Were the study subjects and the setting described in detail? | □ | □X | □ | □ |
| 1. Was the data analysis conducted with sufficient coverage of the identified sample? | □√ | □ | □ | □ |
| 1. Were valid methods used for the identification of the condition? | □√ | □ | □ | □ |
| 1. Was the condition measured in a standard, reliable way for all participants? | □√ | □ | □ | □ |
| 1. Was there appropriate statistical analysis? | □√ | □ | □ | □ |
| 1. Was the response rate adequate, and if not, was the low response rate managed appropriately? | □√ | □ | □ | □ |

Overall appraisal: Include √ □ Exclude □ Seek further info □

Comments (Including reason for exclusion)

**JBI Critical Appraisal Checklist for Studies Reporting Prevalence Data**

Reviewer T.D Date October 24, 2020, G. C

# Author Yibeltal Estemech Year 2020 Record Number 09

|  | Yes | No | Unclear | Not applicable |
| --- | --- | --- | --- | --- |
| 1. Was the sample frame appropriate to address the target population? | □√ | □ | □ | □ |
| 1. Were study participants sampled in an appropriate way? | □√ | □ | □ | □ |
| 1. Was the sample size adequate? | □ | □X | □ | □ |
| 1. Were the study subjects and the setting described in detail? | □ | □X | □ | □ |
| 1. Was the data analysis conducted with sufficient coverage of the identified sample? | □√ | □ | □ | □ |
| 1. Were valid methods used for the identification of the condition? | □√ | □ | □ | □ |
| 1. Was the condition measured in a standard, reliable way for all participants? | □√ | □ | □ | □ |
| 1. Was there appropriate statistical analysis? | □√ | □ | □ | □ |
| 1. Was the response rate adequate, and if not, was the low response rate managed appropriately? | □√ | □ | □ | □ |

Overall appraisal: Include √ □ Exclude □ Seek further info □

Comments (Including reason for exclusion)

**JBI Critical Appraisal Checklist for Studies Reporting Prevalence Data**

Reviewer M.S Date October 24, 2020, G. C

# Author Getinet Shewaseged Year 2017 Record Number 10

|  | Yes | No | Unclear | Not applicable |
| --- | --- | --- | --- | --- |
| 1. Was the sample frame appropriate to address the target population? | □√ | □ | □ | □ |
| 1. Were study participants sampled in an appropriate way? | □√ | □ | □ | □ |
| 1. Was the sample size adequate? | □√ | □ | □ | □ |
| 1. Were the study subjects and the setting described in detail? | □ | □X | □ | □ |
| 1. Was the data analysis conducted with sufficient coverage of the identified sample? | □√ | □ | □ | □ |
| 1. Were valid methods used for the identification of the condition? | □√ | □ | □ | □ |
| 1. Was the condition measured in a standard, reliable way for all participants? | □√ | □ | □ | □ |
| 1. Was there appropriate statistical analysis? | □√ | □ | □ | □ |
| 1. Was the response rate adequate, and if not, was the low response rate managed appropriately? | □√ | □ | □ | □ |

Overall appraisal: Include √ □ Exclude □ Seek further info □

Comments (Including reason for exclusion)

**JBI Critical Appraisal Checklist for Studies Reporting Prevalence Data**

Reviewer M.S Date October 24, 2020, G. C

# Author Getinet Shewaseged Year 2017 Record Number 10

|  | Yes | No | Unclear | Not applicable |
| --- | --- | --- | --- | --- |
| 1. Was the sample frame appropriate to address the target population? | □√ | □ | □ | □ |
| 1. Were study participants sampled in an appropriate way? | □√ | □ | □ | □ |
| 1. Was the sample size adequate? | □√ | □ | □ | □ |
| 1. Were the study subjects and the setting described in detail? | □ | □X | □ | □ |
| 1. Was the data analysis conducted with sufficient coverage of the identified sample? | □√ | □ | □ | □ |
| 1. Were valid methods used for the identification of the condition? | □√ | □ | □ | □ |
| 1. Was the condition measured in a standard, reliable way for all participants? | □√ | □ | □ | □ |
| 1. Was there appropriate statistical analysis? | □√ | □ | □ | □ |
| 1. Was the response rate adequate, and if not, was the low response rate managed appropriately? | □√ | □ | □ | □ |

Overall appraisal: Include √ □ Exclude □ Seek further info □

Comments (Including reason for exclusion)

**JBI Critical Appraisal Checklist for Studies Reporting Prevalence Data**

Reviewer M.S Date October 24, 2020, G. C

# Author Abdulbasit H Year 2015 Record Number 11

|  | Yes | No | Unclear | Not applicable |
| --- | --- | --- | --- | --- |
| 1. Was the sample frame appropriate to address the target population? | □√ | □ | □ | □ |
| 1. Were study participants sampled in an appropriate way? | □√ | □ | □ | □ |
| 1. Was the sample size adequate? | □ | □X | □ | □ |
| 1. Were the study subjects and the setting described in detail? | □ | □X | □ | □ |
| 1. Was the data analysis conducted with sufficient coverage of the identified sample? | □√ | □ | □ | □ |
| 1. Were valid methods used for the identification of the condition? | □√ | □ | □ | □ |
| 1. Was the condition measured in a standard, reliable way for all participants? | □√ | □ | □ | □ |
| 1. Was there appropriate statistical analysis? | □√ | □ | □ | □ |
| 1. Was the response rate adequate, and if not, was the low response rate managed appropriately? | □√ | □ | □ | □ |

Overall appraisal: Include √ □ Exclude □ Seek further info □

Comments (Including reason for exclusion)

**JBI Critical Appraisal Checklist for Studies Reporting Prevalence Data**

Reviewer T.D Date October 24, 2020, G. C

# Author Abdulbasit H Year 2015 Record Number 11

|  | Yes | No | Unclear | Not applicable |
| --- | --- | --- | --- | --- |
| 1. Was the sample frame appropriate to address the target population? | □√ | □ | □ | □ |
| 1. Were study participants sampled in an appropriate way? | □√ | □ | □ | □ |
| 1. Was the sample size adequate? | □ | □X | □ | □ |
| 1. Were the study subjects and the setting described in detail? | □ | □X | □ | □ |
| 1. Was the data analysis conducted with sufficient coverage of the identified sample? | □√ | □ | □ | □ |
| 1. Were valid methods used for the identification of the condition? | □√ | □ | □ | □ |
| 1. Was the condition measured in a standard, reliable way for all participants? | □√ | □ | □ | □ |
| 1. Was there appropriate statistical analysis? | □√ | □ | □ | □ |
| 1. Was the response rate adequate, and if not, was the low response rate managed appropriately? | □√ | □ | □ | □ |

Overall appraisal: Include √ □ Exclude □ Seek further info □

Comments (Including reason for exclusion)

**JBI Critical Appraisal Checklist for Studies Reporting Prevalence Data**

Reviewer M.S Date October 24, 2020, G. C

# Author Mohammed Abdu Year 2020 Record Number 12

|  | Yes | No | Unclear | Not applicable |
| --- | --- | --- | --- | --- |
| 1. Was the sample frame appropriate to address the target population? | □√ | □ | □ | □ |
| 1. Were study participants sampled in an appropriate way? | □√ | □ | □ | □ |
| 1. Was the sample size adequate? | □ | □X | □ | □ |
| 1. Were the study subjects and the setting described in detail? | □ | □X | □ | □ |
| 1. Was the data analysis conducted with sufficient coverage of the identified sample? | □√ | □ | □ | □ |
| 1. Were valid methods used for the identification of the condition? | □√ | □ | □ | □ |
| 1. Was the condition measured in a standard, reliable way for all participants? | □√ | □ | □ | □ |
| 1. Was there appropriate statistical analysis? | □√ | □ | □ | □ |
| 1. Was the response rate adequate, and if not, was the low response rate managed appropriately? | □√ | □ | □ | □ |

Overall appraisal: Include √ □ Exclude □ Seek further info □

Comments (Including reason for exclusion)

**JBI Critical Appraisal Checklist for Studies Reporting Prevalence Data**

Reviewer M.S Date October 24, 2020, G. C

# Author Mohammed Abdu Year 2020 Record Number 12

|  | Yes | No | Unclear | Not applicable |
| --- | --- | --- | --- | --- |
| 1. Was the sample frame appropriate to address the target population? | □√ | □ | □ | □ |
| 1. Were study participants sampled in an appropriate way? | □√ | □ | □ | □ |
| 1. Was the sample size adequate? | □ | □X | □ | □ |
| 1. Were the study subjects and the setting described in detail? | □ | □X | □ | □ |
| 1. Was the data analysis conducted with sufficient coverage of the identified sample? | □√ | □ | □ | □ |
| 1. Were valid methods used for the identification of the condition? | □√ | □ | □ | □ |
| 1. Was the condition measured in a standard, reliable way for all participants? | □√ | □ | □ | □ |
| 1. Was there appropriate statistical analysis? | □√ | □ | □ | □ |
| 1. Was the response rate adequate, and if not, was the low response rate managed appropriately? | □√ | □ | □ | □ |

Overall appraisal: Include √ □ Exclude □ Seek further info □

Comments (Including reason for exclusion)

**JBI Critical Appraisal Checklist for Studies Reporting Prevalence Data**

Reviewer M.S Date October 24, 2020, G. C

# Author Abiyot Asres Year 2020 Record Number 13

|  | Yes | No | Unclear | Not applicable |
| --- | --- | --- | --- | --- |
| 1. Was the sample frame appropriate to address the target population? | □√ | □ | □ | □ |
| 1. Were study participants sampled in an appropriate way? | □√ | □ | □ | □ |
| 1. Was the sample size adequate? | □√ | □ | □ | □ |
| 1. Were the study subjects and the setting described in detail? | □ | □X | □ | □ |
| 1. Was the data analysis conducted with sufficient coverage of the identified sample? | □√ | □ | □ | □ |
| 1. Were valid methods used for the identification of the condition? | □√ | □ | □ | □ |
| 1. Was the condition measured in a standard, reliable way for all participants? | □ | □x | □ | □ |
| 1. Was there appropriate statistical analysis? | □√ | □ | □ | □ |
| 1. Was the response rate adequate, and if not, was the low response rate managed appropriately? | □√ | □x | □ | □ |

Overall appraisal: Include □ Exclude √ □ Seek further info □

Comments (Including reason for exclusion)

**JBI Critical Appraisal Checklist for Studies Reporting Prevalence Data**

Reviewer T.D Date October 24, 2020, G. C

# Author Abiyot Asres Year 2020 Record Number 13

|  | Yes | No | Unclear | Not applicable |
| --- | --- | --- | --- | --- |
| 1. Was the sample frame appropriate to address the target population? | □√ | □ | □ | □ |
| 1. Were study participants sampled in an appropriate way? | □√ | □ | □ | □ |
| 1. Was the sample size adequate? | □√ | □ | □ | □ |
| 1. Were the study subjects and the setting described in detail? | □ | □X | □ | □ |
| 1. Was the data analysis conducted with sufficient coverage of the identified sample? | □√ | □ | □ | □ |
| 1. Were valid methods used for the identification of the condition? | □√ | □ | □ | □ |
| 1. Was the condition measured in a standard, reliable way for all participants? | □ | □x | □ | □ |
| 1. Was there appropriate statistical analysis? | □√ | □ | □ | □ |
| 1. Was the response rate adequate, and if not, was the low response rate managed appropriately? | □√ | □x | □ | □ |

Overall appraisal: Include □ Exclude √ □ Seek further info □

Comments (Including reason for exclusion)

**JBI Critical Appraisal Checklist for Studies Reporting Prevalence Data**

Reviewer M.S Date October 24, 2020, G. C

# Author Senedu Year 2020 Record Number 14

|  | Yes | No | Unclear | Not applicable |
| --- | --- | --- | --- | --- |
| 1. Was the sample frame appropriate to address the target population? | □√ | □ | □ | □ |
| 1. Were study participants sampled in an appropriate way? | □√ | □ | □ | □ |
| 1. Was the sample size adequate? | □√ | □ | □ | □ |
| 1. Were the study subjects and the setting described in detail? | □ | □X | □ | □ |
| 1. Was the data analysis conducted with sufficient coverage of the identified sample? | □√ | □ | □ | □ |
| 1. Were valid methods used for the identification of the condition? | □√ | □ | □ | □ |
| 1. Was the condition measured in a standard, reliable way for all participants? | □ | □x | □ | □ |
| 1. Was there appropriate statistical analysis? | □√ | □ | □ | □ |
| 1. Was the response rate adequate, and if not, was the low response rate managed appropriately? | □√ | □x | □ | □ |

Overall appraisal: Include □ Exclude √ □ Seek further info □

Comments (Including reason for exclusion)

**JBI Critical Appraisal Checklist for Studies Reporting Prevalence Data**

Reviewer T.D Date October 24, 2020, G. C

# Author Senedu Year 2020 Record Number 14

|  | Yes | No | Unclear | Not applicable |
| --- | --- | --- | --- | --- |
| 1. Was the sample frame appropriate to address the target population? | □√ | □ | □ | □ |
| 1. Were study participants sampled in an appropriate way? | □√ | □ | □ | □ |
| 1. Was the sample size adequate? | □√ | □ | □ | □ |
| 1. Were the study subjects and the setting described in detail? | □ | □X | □ | □ |
| 1. Was the data analysis conducted with sufficient coverage of the identified sample? | □√ | □ | □ | □ |
| 1. Were valid methods used for the identification of the condition? | □√ | □ | □ | □ |
| 1. Was the condition measured in a standard, reliable way for all participants? | □ | □x | □ | □ |
| 1. Was there appropriate statistical analysis? | □√ | □ | □ | □ |
| 1. Was the response rate adequate, and if not, was the low response rate managed appropriately? | □√ | □x | □ | □ |

Overall appraisal: Include □ Exclude √ □ Seek further info □

Comments (Including reason for exclusion)

**JBI Critical Appraisal Checklist for Studies Reporting Prevalence Data**

Reviewer M.S Date October 24, 2020, G. C

# Author Fentabil Year 2019 Record Number 15

|  | Yes | No | Unclear | Not applicable |
| --- | --- | --- | --- | --- |
| 1. Was the sample frame appropriate to address the target population? | □√ | □ | □ | □ |
| 1. Were study participants sampled in an appropriate way? | □√ | □ | □ | □ |
| 1. Was the sample size adequate? | □√ | □ | □ | □ |
| 1. Were the study subjects and the setting described in detail? | □ | □X | □ | □ |
| 1. Was the data analysis conducted with sufficient coverage of the identified sample? | □√ | □ | □ | □ |
| 1. Were valid methods used for the identification of the condition? | □√ | □ | □ | □ |
| 1. Was the condition measured in a standard, reliable way for all participants? | □ | □x | □ | □ |
| 1. Was there appropriate statistical analysis? | □√ | □ | □ | □ |
| 1. Was the response rate adequate, and if not, was the low response rate managed appropriately? | □√ | □x | □ | □ |

Overall appraisal: Include □ Exclude √ □ Seek further info □

Comments (Including reason for exclusion)

**JBI Critical Appraisal Checklist for Studies Reporting Prevalence Data**

Reviewer T.D Date October 24, 2020, G. C

# Author Fentabil Year 2019 Record Number 15

|  | Yes | No | Unclear | Not applicable |
| --- | --- | --- | --- | --- |
| 1. Was the sample frame appropriate to address the target population? | □√ | □ | □ | □ |
| 1. Were study participants sampled in an appropriate way? | □√ | □ | □ | □ |
| 1. Was the sample size adequate? | □√ | □ | □ | □ |
| 1. Were the study subjects and the setting described in detail? | □ | □X | □ | □ |
| 1. Was the data analysis conducted with sufficient coverage of the identified sample? | □√ | □ | □ | □ |
| 1. Were valid methods used for the identification of the condition? | □√ | □ | □ | □ |
| 1. Was the condition measured in a standard, reliable way for all participants? | □ | □x | □ | □ |
| 1. Was there appropriate statistical analysis? | □√ | □ | □ | □ |
| 1. Was the response rate adequate, and if not, was the low response rate managed appropriately? | □√ | □x | □ | □ |

Overall appraisal: Include □ Exclude √ □ Seek further info □

Comments (Including reason for exclusion)

**JBI Critical Appraisal Checklist for Studies Reporting Prevalence Data**

Reviewer M.S Date October 24, 2020, G. C

# Author Abdullah Year 2018 Record Number 16

|  | Yes | No | Unclear | Not applicable |
| --- | --- | --- | --- | --- |
| 1. Was the sample frame appropriate to address the target population? | □√ | □ | □ | □ |
| 1. Were study participants sampled in an appropriate way? | □ | □x | □ | □ |
| 1. Was the sample size adequate? | □√ | □ | □ | □ |
| 1. Were the study subjects and the setting described in detail? | □ | □X | □ | □ |
| 1. Was the data analysis conducted with sufficient coverage of the identified sample? | □√ | □ | □ | □ |
| 1. Were valid methods used for the identification of the condition? | □√ | □ | □ | □ |
| 1. Was the condition measured in a standard, reliable way for all participants? | □ | □x | □ | □ |
| 1. Was there appropriate statistical analysis? | □√ | □ | □ | □ |
| 1. Was the response rate adequate, and if not, was the low response rate managed appropriately? | □√ | □x | □ | □ |

Overall appraisal: Include □ Exclude √ □ Seek further info □

Comments (Including reason for exclusion)

Reviewer T.D Date October 24, 2020, G. C

# Author Abdullah Year 2018 Record Number 16

|  | Yes | No | Unclear | Not applicable |
| --- | --- | --- | --- | --- |
| 1. Was the sample frame appropriate to address the target population? | □√ | □ | □ | □ |
| 1. Were study participants sampled in an appropriate way? | □ | □x | □ | □ |
| 1. Was the sample size adequate? | □√ | □ | □ | □ |
| 1. Were the study subjects and the setting described in detail? | □ | □X | □ | □ |
| 1. Was the data analysis conducted with sufficient coverage of the identified sample? | □√ | □ | □ | □ |
| 1. Were valid methods used for the identification of the condition? | □√ | □ | □ | □ |
| 1. Was the condition measured in a standard, reliable way for all participants? | □ | □x | □ | □ |
| 1. Was there appropriate statistical analysis? | □ | □x | □ | □ |
| 1. Was the response rate adequate, and if not, was the low response rate managed appropriately? | □√ | □x | □ | □ |

Overall appraisal: Include □ Exclude √ □ Seek further info □

Comments (Including reason for exclusion)

**JBI Critical Appraisal Checklist for Studies Reporting Prevalence Data**

Reviewer M.S Date October 24, 2020, G. C

# Author Awel Husen Year 2012 Record Number 17

|  | Yes | No | Unclear | Not applicable |
| --- | --- | --- | --- | --- |
| 1. Was the sample frame appropriate to address the target population? | □√ | □ | □ | □ |
| 1. Were study participants sampled in an appropriate way? | □ | □x | □ | □ |
| 1. Was the sample size adequate? | □ | □x | □ | □ |
| 1. Were the study subjects and the setting described in detail? | □ | □X | □ | □ |
| 1. Was the data analysis conducted with sufficient coverage of the identified sample? | □√ | □ | □ | □ |
| 1. Were valid methods used for the identification of the condition? | □√ | □ | □ | □ |
| 1. Was the condition measured in a standard, reliable way for all participants? | □ | □x | □ | □ |
| 1. Was there appropriate statistical analysis? | □√ | □ | □ | □ |
| 1. Was the response rate adequate, and if not, was the low response rate managed appropriately? | □√ | □x | □ | □ |

Overall appraisal: Include □ Exclude √ □ Seek further info □

Comments (Including reason for exclusion)

**JBI Critical Appraisal Checklist for Studies Reporting Prevalence Data**

Reviewer M.S Date October 24, 2020, G. C

# Author Awel Husen Year 2012 Record Number 17

|  | Yes | No | Unclear | Not applicable |
| --- | --- | --- | --- | --- |
| 1. Was the sample frame appropriate to address the target population? | □√ | □ | □ | □ |
| 1. Were study participants sampled in an appropriate way? | □ | □x | □ | □ |
| 1. Was the sample size adequate? | □ | □x | □ | □ |
| 1. Were the study subjects and the setting described in detail? | □ | □X | □ | □ |
| 1. Was the data analysis conducted with sufficient coverage of the identified sample? | □√ | □ | □ | □ |
| 1. Were valid methods used for the identification of the condition? | □√ | □ | □ | □ |
| 1. Was the condition measured in a standard, reliable way for all participants? | □ | □x | □ | □ |
| 1. Was there appropriate statistical analysis? | □√ | □ | □ | □ |
| 1. Was the response rate adequate, and if not, was the low response rate managed appropriately? | □√ | □ | □ | □ |

Overall appraisal: Include □ Exclude √ □ Seek further info □

Comments (Including reason for exclusion)
